# Annotations

**Published:** 1905-02-04

**Authors:** 


### ANNOTATIONS.

### Illness of Princess Victoria.

THE announcement, on Tuesday morning, of the serious illness of Princess Victoria from appendicitis, was a painful surprise to all classes of the community. It was understood that her Royal Highness was out of health, but it was not known that the Royal Family had any reason for grave concern. A fortnight ago she was more than usually unwell, and Sir Francis Laking and Sir Frederick Treves were summoned to Sandringham. It was then decided that an operation was advisable, and, the date being fixed, Princess Victoria came to London last Friday in order to prepare for it. The first bulletin, issued from Buckingham Palace at 11 A.M. on Tuesday, conveyed the welcome intelligence that the circumstances of the operation were favourable, that the Princess bore the operation well, and was progressing satisfactorily. Subsequent bulletins have been equally reassuring, but there will, of course, be occasion for solicitude until the patient is pronounced convalescent. Meanwhile, the event has been the means of affording yet another striking illustration of the desire of the King and Queen to avoid provoking needless apprehension on the part of the public, which would certainly have been rife if the nature of the Royal patient's illness had been disclosed some days previous to the operation. It has also, while emphasising their Majesties' thoughtful consideration for their subjects, enabled the latter to demonstrate their sympathy for Princess Victoria in her illness, and for the members of the Royal Family in their anxiety. The knowledge that in all quarters of the Empire the fervent wish is entertained for a speedy and complete recovery of their daughter, cannot but tend to relieve the suspense of their Majesties during the period which must elapse before she is able to resume her place in the family circle.

### The Dispensing of Poisons.

A STORY, if not frequent, at least familiar, was related in a coroner's court in London a short while ago. The victim, whose death formed the subject of inquiry, was a woman who had been under treatment for sciatica, and who had received from her medical adviser a bottle of medicine for internal administration, and a second bottle containing liniment of belladonna and chloroform. This latter, while labelled "Not to be taken—for external application only," was an ordinary bottle, destitute of any physical characters calculated to warn any person handling it that it contained dangerous or poisonous materials. The next act in the tragedy was a mistake made by a friend of the deceased, and the administration to the latter of a fatal dose of the poisonous liniment. As has occurred before, the one bottle was mistaken for the other with consequences disastrous to all concerned. Had the event been an isolated one, severe remarks might justifiably be made on the person immediately responsible for the fatal issue. But, time and again, experience has shown that no amount of dramatic quality in the label will prevent persons who are unaccustomed to deal with medicines from confusing remedies intended for external application with those ordered for internal use. It therefore rests with those who dispense the former to take such precautions as will readily excite the attention even of stupid or careless persons. Bottles to secure this end are readily obtained, and the need for them has been demonstrated over and over again. The law does not compel medical men to use such contrivances, but an adequate sense of moral responsibility ought to do so. We have said before, and we have no hesitation in repeating, that all the precautions which are demanded of the pharmacist in the storage and sale of poisons ought to be adopted by every medical practitioner who supplies medicines to his patients. The need for these exists in the hospital dispensary and in the private surgery as it also exists in the shop of the chemist and druggist. Neglect of these precautions means risk to human life, and when unfortunately that risk is realised the responsibility cannot be wholly transferred to the shoulders of the later actors in the tragedy. In dealing with edged tools it is necessary to recognise the existence both of the ignorant and the foolish.

### The Organisation of Public Health Statistics.

A SEVERE and sweeping criticism of the existing methods of recording and publishing statistical data bearing on public health has recently been presented to the Royal Statistical Society by Dr. Reginald Dudfield. Speaking generally, it was urged that under present arrangements there is a deficiency of organisation and co-ordination in the collection and publication of the official statistics, and that, the reports issued by various independent authorities not being constructed on a common plan, efficient comparison and analysis is impossible. Another comment referred to the long delay which so frequently attends the issue of statistics dealing with questions of the public health. In the Registrar-General's annual report two specific deficiencies were noted—namely, the absence of information regarding the numbers of persons at different ages and the want of analysis of the causes of death according to the sex and age of the deceased. It was also urged that there is a general failure to utilise the information afforded by the poor-law records, by the returns from hospitals and benefit societies, and by the notification of infectious diseases, all of which bear upon the spread of disease and the general health of the nation. The certification both of births and deaths was pronounced to be unsatisfactory and in urgent need of reform. Apart from many improvements regarding details, the author advocated the establishment of a central statistical office independent of existing Government departments and charged with the duty of co-ordinating the work of all those engaged in the preparation of national vital statistics. The advisory committee recommended by the Committee on Physical Deterioration was suggested as a suitable body for this purpose, and to it should be entrusted the compilation of hospital returns and of sickness and benefit societies, as well as a register of sickness and the conduct of an anthropometric survey. There will doubtless be general assent to the principal counts in this criticism. Simplicity, comprehensiveness, and promptness of issue are highly to be desired in statistics bearing on the public health, and even those who delight to dwell on the misleading influence of "figures" will agree that accurate returns are less pernicious than inaccurate ones.

